# The Training Effect of Early Intervention with a Hybrid Assistive Limb after Total Knee Arthroplasty

**DOI:** 10.1155/2017/6912706

**Published:** 2017-08-10

**Authors:** Takashi Fukaya, Hirotaka Mutsuzaki, Kenichi Yoshikawa, Ayumu Sano, Masafumi Mizukami, Masashi Yamazaki

**Affiliations:** ^1^Department of Physical Therapy, Faculty of Health Sciences, Tsukuba International University, 6-8-33 Manabe, Tsuchiura, Ibaraki 300-0051, Japan; ^2^Department of Orthopaedic Surgery, Ibaraki Prefectural University of Health Sciences, 4669-2 Ami, Ami-machi, Inashiki-gun, Ibaraki 300-0394, Japan; ^3^Department of Physical Therapy, Ibaraki Prefectural University of Health Sciences Hospital, 4773 Ami, Ami-machi, Inashiki-gun, Ibaraki 300-0331, Japan; ^4^Department of Physical Therapy, Ibaraki Prefectural University of Health Sciences, 4669-2 Ami, Ami-machi, Inashiki-gun, Ibaraki 300-0394, Japan; ^5^Department of Orthopaedic Surgery, University of Tsukuba, 1-1-1 Tennodai, Tsukuba, Ibaraki 305-8575, Japan

## Abstract

After total knee arthroplasty (TKA), it is important for patients to show early improvements in knee joint function and walking to regain independence in performing the activities of daily life. We conducted for 4 weeks an intervention one week after TKA using a hybrid assistive limb (HAL: unilateral leg type) as an exoskeleton robotic device to facilitate knee joint function and walking. The intervention improved the range of knee extension movement safely and without pain compared to preoperation. In addition, we found that training with the HAL improved walking ability, speed, and rate, as well as the time taken to perform the timed up and go (TUG) test compared to preoperation. The strength of the quadriceps muscle did not improve with training; however, the patient was able to induce a knee extensor moment during the initial stance phase, as measured by kinetics and kinematics, and these actions could be performed without pain. HAL training soon after TKA improved knee joint function in a 76-year-old patient who presented with OA of the knee. The improvements in knee extension lag and knee extensor moment allowed the patient to walk without pain and regain ADL in comparison with preoperation.

## 1. Introduction

Patients with knee osteoarthritis (OA) present with pain and disrupted gait during walking, a restricted range of motion (ROM), and muscle weakness. In patients with severe knee OA, knee extension during the midstance phase is decreased, and patients do not show the typical “double knee action,” that is, normal gait that requires the knee to undergo two knee flexions/extensions during one cycle [1]. Because the ROM is restricted along with pain during walking, the knee extensor muscle weakens, and patients show a decrease in the knee extensor moment exerted during the stance phase [2]. Walking pattern, speed, rate, and the number of steps also change in these patients as they try to minimize their pain [3–5]. Because such knee joint dysfunction limits the walking and walking ability, total knee arthroplasty (TKA) is commonly performed in patients with knee OA to improve joint dysfunction.

Proper knee joint function (i.e., walking ability, muscle strength, and full ROM) is important for the walking and walking ability and there is a need for patients to recover quickly after surgery. However, previous studies showed that the ROM of the knee joint is restricted until about 12 months after TKA [6, 7]. Smoothness of the knee joint movement and enough mobility are necessary to accomplish walking; therefore, appropriate treatment strategies need to be employed to recover the joint movement necessary for the walking and walking ability. Hybrid assistive limb (HAL, Cyberdyne Inc., Tsukuba, Japan) technology is reported to be effective in the functional recovery of various locomotor disabilities, such as disrupted walking/gait, restricted ROM, and muscle weakness [8, 9]. The HAL perceives the subject's muscle activity through bioelectric signals and ground-reaction-force signals caused by the subject's shift in weight. Accordingly, exercise therapy with a HAL can facilitate proper gait in the patient in a voluntary manner. Studies have shown improvements in knee joint function with a single-joint HAL during the early postoperative period after TKA in a patient with knee OA [10]; however, the single-joint HAL has no ground-reaction-force sensor and was used solely for improving knee joint function. In recent years, there have also been reports of the use of a HAL for exercise therapy in patients following subacute stroke [11]. To date, there have been no reports of the kinematic and kinetic outcomes of walking and walking ability, or improvements in knee joint function using the HAL after TKA in a patient with knee OA. Therefore, we explored the use of a HAL in conjunction with exercise therapy for 4 weeks after TKA in a patient who presented with knee OA. We examined whether the HAL could aid in the recovery of joint movement, walking ability, and walking patterns, as well as changes to the knee joint moment during walking in this patient.

## 2. Case Presentation

### 2.1. Patient

The patient was a 76-year-old male who underwent right TKA for grade 4 knee OA (Kellgren-Lawrence scale). The subject's knee pain started 9 years ago, and he observed its progress while taking an internal medicine. Last year, knee orthotic with struts on both sides was prescribed, but because activity of daily life became difficult, he was hospitalized for surgical purposes. The knee pain was strong when ascending and descending stairs, and walking on a flat ground was possible using a cane for about 500 meters.

### 2.2. Pre- and Postoperative Evaluation

Physical evaluations were conducted before surgery. The subject was instructed to walk along a 10 m walkway at his preferred speed, and the following variables were calculated: walking speed, walking rate, number of steps, and step length. We then conducted a timed up and go (TUG) test, which measures the time it takes the patient to rise from a chair, walk 3 m, turn around, walk back to the chair, and sit down. The muscular strength of the knee joint was measured with an isometric mode using Biodex System 4. The fixed angle of the knee joint was set up to 60 degrees of knee flexion and knee joint flexion and extension exercise was conducted in 3 sets every five seconds, and we also performed both active and passive ROMs for extension, and flexion of the knee joint was measured using a goniometer by a physical therapist. For gait analysis, a motion analysis system (Vicon Nexus, Oxford, UK) and floor-mounted force plates (Kistler Instruments, Winterthur, Switzerland) were used to calculate knee joint movement and knee joint moment. The kinematic data were obtained at 200 Hz using an 8-camera motion analysis system, and the kinetics data were recorded at 1200 Hz. Motion was analyzed using the Plug-In-Gait model, and 16 reflective markers, 9.5 mm in diameter, were placed directly over the following bilateral anatomical landmarks: anterior and posterior superior iliac spines, lateral center of the thigh, lateral femoral epicondyle, lateral center of the shank, lateral malleoli, calcaneus, and the top of the foot at the base of the second metatarsal.

Knee joint movement was normalized to the values at 100% through one gait cycle (foot strike to next foot strike = 100%) using spline interpolation. Knee joint moments, as determined by the force plate data, were normalized to the values at 60% through the stance phase (foot strike to toe-off = 60%). The joint angles during the patient's gait cycle were calculated using the joint coordinate system approach described by Grood and Suntay [12]. Calculations of the kinetics data used the inverse dynamics techniques. Body segment parameters for kinetics data calculations used coefficients specific to the Japanese population, as reported by Okada et al. [13]. In addition, the stance phase of the gait cycle was defined using five sections according to Perry [14], as follows: initial contact (IC = 0%), loading response (LR = 12%), midstance (MSt = 31%), terminal stance (TSt = 50%), and preswing (PSw = 60%).

To verify the immediate effect of HAL training, data were collected using these procedures before and at 5 weeks of HAL training after surgery.

### 2.3. Surgery of the Knee Joint

The patient was given general anesthesia. No tourniquet was used for the surgery. A medial parapatellar approach was used through a midline skin incision. An intramedullary alignment rod was used for femoral cutting and an extramedullary guide system for tibial cutting. The implants used were the NexGen CR (Zimmer, Warsaw, IN, USA), with the CR-Flex Porous Femoral and Trabecular Metal Monoblock Tibia for the femoral and tibial component, respectively. The patella was not replaced, the posterior cruciate ligament was retained, and all components were fixed without cement. Following surgery, the patient underwent intravenous prophylactic antibiotic therapy consisting of 1 g cefazolin every 12 h for 3 days. A foot pump (Novamedix A-V Impulse System; Kobayashi Medical, Osaka, Japan) and antiembolism stockings (Ansilk®; ALCARE, Tokyo, Japan) were used for thromboembolic prophylaxis [15, 16]. Sutures were removed at 2 weeks after surgery. As part of postoperative care, continuous passive movement (CPM) was started on postoperative day 3, and standing and full-weight-bearing walking were allowed one day after surgery.

### 2.4. HAL Training Protocol

The HAL (unilateral leg type) was a medium-sized FL05 (Cyberdyne Inc., Tsukuba, Japan). The patient changed into a jersey, and a physical therapist measured the patient's body weight, femoral and shank length, hip width, and vital signs before setting the HAL. After measuring the maximum flexion angle and extension angle of the knee joint before the intervention, the angle was set so as not to overassist the HAL assist angle. Electrodes were attached to the patient's rectus femoris, gluteus maximus, vastus lateralis, and biceps femoris, each identified via palpation. The HAL was set with the patient seated. Before HAL training, the patient underwent bilateral plain radiography performed by a radiology technician using Rosenberg's 45° flexion load-bearing view and one orthopedic surgeon interpreted the radiograph of the patients. By the result of the radiograph, we confirmed that the patient was not suffering from osteoporosis or deformation to the lower extremity. In addition, we confirmed whether dizziness occurs by interrogation. After the subject confirmed the waveform of electromyogram (EMG) while extending the knee joint with HAL, we checked whether muscles contraction occurs with regular timing during walking.

**Figure 1:**
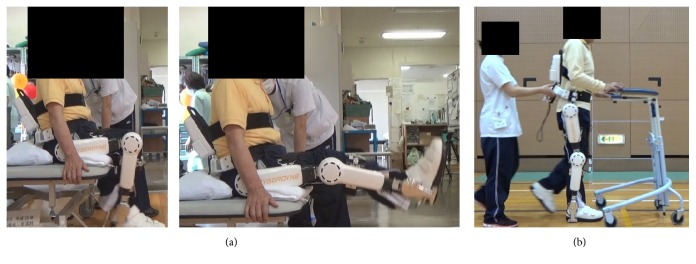
Photo of the hybrid assistive limb (HAL) on the patient (a). The patient is a 76-year-old male who underwent right TKA for grade 4 knee OA (Kellgren-Lawrence scale). Photo of the HAL training during walking (b).

HAL training was performed 12 times over 4 weeks, beginning one week after surgery (week 1 to week 5; Figure 1). Training incorporated active extension and flexion exercises of the knee joint while adjusting the knee assisted level with HAL in a seated position and gait exercises at a comfortable walking speed, each test for about 10 min. The subject walked round about 45-meter walking path using a U-shaped walker. The assist level and balance of hip and knee joint were adjusted while checking the state of walking. During training, we monitored the patient's pain levels and lowered the intensity to avoid pain. In addition, while asking the subject to confirm the waveform of EMG, we instructed the subject to generate a similar waveform when performing active extension and flexion exercises of the knee joint. During active extension and flexion exercises of the knee joint, we fed back the waveform of EMG to the subject. When walking, we instructed the patient not to be in a forward tilted posture. Walking exercise took place with a break as appropriate. For orthopedic treatment, an internal analgesic was taken, and wound treatment was completed in 2 weeks. In addition to HAL training, joint mobilization was implemented by a physical therapist, and after training, the subject cooled the affected area.

### 2.5. Postoperative Outcomes

**Figure 2:**
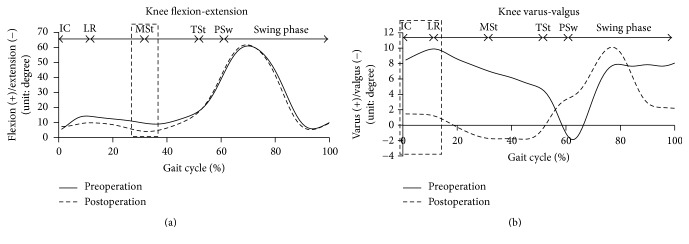
Kinematics of the gait analysis, pre- and postoperative. (a) represents knee flexion-extension and (b) represents knee varus-valgus during gait cycle. The part surrounded by the square in the figure shows the feature of knee kinematics change.

**Figure 3:**
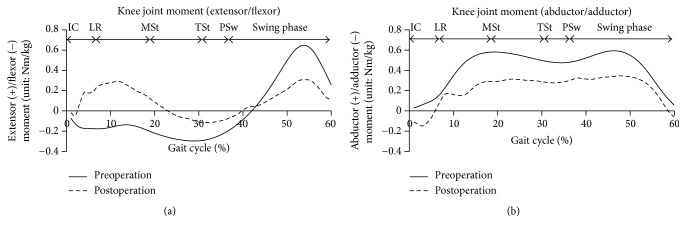
Pre- and postoperative knee joint moments. (a) represents knee extensor-flexor and (b) represents knee adductor-abductor moment during gait cycle.

The walking speed, walking rate, and TUG tests improved after surgery (Table 1). The patient's ROM also improved, but muscle strength of the knee extensor group did not sufficiently recover within the 5-week postsurgical period (Table 2). The pre- and postoperative kinematic gait results are shown in Figure 2. The restricted knee extension seen preoperatively at MSt was improved after treatment (Figure 2(a)). The knee varus thrust in the frontal plane from IC to LR disappeared after surgery (Figure 2(b)). The knee abductor moment decreased after surgery, and the patient could exert a knee extensor moment from IC to MSt after the operation (Figure 3).

## 3. Discussion

In this case study, the patient showed considerable improvement in knee function and in attaining the activity of daily life (ADL) after HAL-directed exercise therapy. We found that walking parameters, such as walking speed, rate, and TUG, all improved within 5 weeks. In addition, there were improvements in gait and ROMs for flexion and extension, as determined by kinematics and kinetics analyses. Although the muscle strength of the knee extensor group did not improve, the patient showed improved knee extension lag, which was thought to be the result of quadriceps weakness before the surgery. In addition, the improved ROM from the training with the HAL could be achieved without pain, and this is an important aspect that often hinders the rate of improvement in patients after surgery. We also performed training with CPM in the hospital room; however, others have reported that neither active nor passive ROMs improve with CPM [17]. In the past, the ROM after TKA was restricted for about 12 months; in addition, the ROM of patients decreased during a period of 1 month after TKA [6, 7]. However, we show that early intervention with the HAL can provide early positive benefit to the patient's ROM within 5 weeks. Also, the nonoperation side of the knee joint showed a drastic change in the range of motion after surgery. Since pain on the operation side improved and the operation side became a supporting leg after the surgery, the pain on the nonoperative side also improved. Therefore, the effect of HAL training can spread not only to the operation side but also to the nonoperation side.

Improvements in walking after TKA are necessary for the patient to be able to perform ADL. Because the HAL detects bioelectric signals of muscle activity and the ground-reaction-force signals during the stance phase of walking, this technology supports the patient's muscle activity in a way that is specific to the patient [11]. The improvement of TUG that is one of the indexes of movement instability appears to be the effective intervention regarding fall prevention; in addition, the increase of the walking speed and rate is likely to affect the improvement of the walking efficiency. We believe that the improvement in walking speed and rate was a consequence of both the reconstruction by TKA and the early intervention with the HAL to induce movement. Actually, our result showed an improvement in the walking speed earlier in comparison with a general treatment process of TKA [18]. Although we did not measure an improvement in muscle strength, the HAL allowed the patient to start moving and acquire the ADL in a safe manner and without pain.

HAL training improved the function of the knee joint not only during isometric knee extension in ROM tests, but also in knee extension at MSt during walking and extension lag. Proper knee extension is vital, as it reduces the biomechanical load at the knee joint at MSt during walking and will support the subject's weight as it is transferred during the knee extension lag when walking. We also found that TKA improved the knee joint extensor moment during the initial stance phase. Patients with severe knee OA find it difficult to exert this knee joint extensor moment because of quadriceps muscle weakness and pain. Although the quadriceps muscle strength did not improve in our patient, he was able to acquire this knee joint extensor moment without pain; we suggest that training with the HAL facilitated this muscular function without pain.

Yoshioka et al. [10] reported that early intervention with HAL after TKA is anticipated to reduce medical costs, as hospital stays and the nursing care may reduce by early recovery of knee function. Our results also showed that HAL training soon after TKA may become useful for knee joint function. In addition, early intervention with the HAL is an effective method to recover knee joint function during walking. This study was the result of a single case study; therefore, a prospective comparative study is necessary to ascertain the effect of training with the HAL after TKA before we can recommend its use in all patients recovering from knee surgery.

In conclusion, HAL training soon after TKA improved knee joint function in a 76-year-old patient who presented with OA of the knee. The improvements in knee extension range and knee extensor moment allowed the patient to walk without pain and regain ADL in comparison with preoperation.

## Figures and Tables

**Table 1:** Pre- and postoperative walking speed, walking rate, and timed up and go (TUG) tests.

	Preoperation	Postoperation
	Average ± SD	Average ± SD
Walking velocity (m/min)	72.29 ± 0.16	76.92 ± 0.41
Steps of 10-meter walks	16.00 ± 0.00	16.00 ± 1.00
Walking rate (steps/min)	115.64 ± 2.23	123.00 ± 3.17
Step length (m)	0.63 ± 0.00	0.63 ± 0.04
Timed up and go (sec)	7.46 ± 0.18	5.92 ± 0.36

**Table 2:** Pre- and postoperative range of motion (ROM), muscle strength, and VAS.

	Preoperation		Postoperation	
	Operation side	Nonoperation side	Operation side	Nonoperation side
ROM of knee flexion (°), passive/active	120/120	125/120	125/120	140/140
ROM of knee extension (°), passive/active	−5/−5	−5/−5	0/0	−5/−5
Knee extensor muscle strength (Nm)	170.9	172.6	119.3	154.9
Knee flexor muscle strength (Nm)	52.9	59.6	60.0	68.3
VAS (visual analog scale)	14	—	0	—

## References

[1] Bytyqi D., Shabani B., Lustig S., Cheze L., Karahoda Gjurgjeala N., Neyret P. (2014). Gait knee kinematic alterations in medial osteoarthritis: Three dimensional assessment. *International Orthopaedics*.

[2] Fukaya T., Mutsuzaki H., Okubo T., Mori K., Wadano Y. (2016). Relationships between the center of pressure and the movements of the ankle and knee joints during the stance phase in patients with severe medial knee osteoarthritis. *The Knee*.

[3] Mündermann A., Dyrby C. O., Hurwitz D. E., Sharma L., Andriacchi T. P. (2004). Potential strategies to reduce medial compartment loading in patients with knee osteoarthritis of varying severity: reduced walking speed. *Arthritis and Rheumatism*.

[4] Debi R., Mor A., Segal G. (2012). Differences in gait pattern parameters between medial and anterior knee pain in patients with osteoarthritis of the knee. *Clinical Biomechanics*.

[5] Messier S. P. (1994). Osteoarthritis of the knee and associated factors of age and obesity: Effects on gait. *Medicine and Science in Sports and Exercise*.

[6] Zhou Z., Yew K. S. A., Arul E. (2015). Recovery in knee range of motion reaches a plateau by 12 months after total knee arthroplasty. *Knee Surgery, Sports Traumatology, Arthroscopy*.

[7] Mizner R. L., Petterson S. C., Snyder-Mackler L. (2005). Quadriceps strength and the time course of functional recovery after total knee arthroplasty. *The Journal of Orthopaedic and Sports Physical Therapy*.

[8] Kubota S., Nakata Y., Eguchi K. (2013). Feasibility of rehabilitation training with a newly developed wearable robot for patients with limited mobility. *Archives of Physical Medicine and Rehabilitation*.

[9] Goto K., Morishita T., Kamada S. (2016). Feasibility of rehabilitation using the single-joint hybrid assistive limb to facilitate early recovery following total knee arthroplasty: a pilot study. *Assistive Technology*.

[10] Yoshioka T., Sugaya H., Kubota S. (2016). Knee-extension training with a single-joint hybrid assistive limb during the early postoperative period after total knee arthroplasty in a patient with osteoarthritis. *Case Reports in Orthopedics*.

[11] Mizukami M., Yoshikawa K., Kawamoto H. (2017). Gait training of subacute stroke patients using a hybrid assistive limb: a pilot study. *Disability and Rehabilitation: Assistive Technology*.

[12] Grood E. S., Suntay W. J. (1983). A joint coordinate system for the clinical description of three-dimensional motions: application to the knee. *Journal of Biomechanical Engineering*.

[13] Okada H., Ae M., Fujii N., Morioka Y. (1996). Body segment inertia properties of Japanese elderly. *Biomechanisms*.

[14] Perry J. (1992). *Gait Analysis: Normal and Pathological Function*.

[15] Morris R. J., Woodcock J. P. (2004). Evidence-based compression: prevention of stasis and deep vein thrombosis. *Annals of Surgery*.

[16] Stannard J. P., Harris R. M., Bucknell A. L., Cossi A., Ward J., Arrington E. D. (1996). Prophylaxis of deep venous thrombosis after total hip arthroplasty by using intermittent compression of the plantar venous plexus. *American Journal of Orthopedics*.

[17] Mistry J. B., Elmallah R. D., Bhave A. (2016). Rehabilitative guidelines after total knee arthroplasty: a review. *Journal of Knee Surgery*.

[18] Shimada N., Deie M., Hirata K. (2016). Courses of change in knee adduction moment and lateral thrust differ up to 1 year after TKA. *Knee Surgery, Sports Traumatology, Arthroscopy*.

